# Using residual regressions to quantify and map signal leakage in genomic prediction

**DOI:** 10.1186/s12711-023-00830-1

**Published:** 2023-08-07

**Authors:** Bruno D. Valente, Gustavo de los Campos, Alexander Grueneberg, Ching-Yi Chen, Roger Ros-Freixedes, William O. Herring

**Affiliations:** 1 The Pig Improvement Company, Genus Plc, Hendersonville, TN USA; 2 Department of Epidemiology and Biostatistics, Michigan State University, East Lansing, MI USA; 3 Department of Statistics and Probability, Michigan State University, East Lansing, MI USA; 4 Institute for Quantitative Health Science and Engineering, Michigan State University, East Lansing, MI USA; 5 The Roslin Institute and Royal (Dick) School of Veterinary Studies, The University of Edinburgh, Easter Bush, Midlothian, Scotland, UK; 6 Departament de Ciència Animal, Universitat de Lleida-Agrotecnio-CERCA Center, Lleida, Spain

## Abstract

**Background:**

Most genomic prediction applications in animal breeding use genotypes with tens of thousands of single nucleotide polymorphisms (SNPs). However, modern sequencing technologies and imputation algorithms can generate ultra-high-density genotypes (including millions of SNPs) at an affordable cost. Empirical studies have not produced clear evidence that using ultra-high-density genotypes can significantly improve prediction accuracy. However, (whole-genome) prediction accuracy is not very informative about the ability of a model to capture the genetic signals from specific genomic regions. To address this problem, we propose a simple methodology that detects chromosome regions for which a specific model (e.g., single-step genomic best linear unbiased prediction (ssGBLUP)) may fail to fully capture the genetic signal present in such segments—a phenomenon that we refer to as signal leakage. We propose to detect regions with evidence of signal leakage by testing the association of residuals from a pedigree or a genomic model with SNP genotypes. We discuss how this approach can be used to map regions with signals that are poorly captured by a model and to identify strategies to fix those problems (e.g., using a different prior or increasing marker density). Finally, we explored the proposed approach to scan for signal leakage of different models (pedigree-based, ssGBLUP, and various Bayesian models) applied to growth-related phenotypes (average daily gain and backfat thickness) in pigs.

**Results:**

We report widespread evidence of signal leakage for pedigree-based models. Including a percentage of animals with SNP data in ssGBLUP reduced the extent of signal leakage. However, local peaks of missed signals remained in some regions, even when all animals were genotyped. Using variable selection priors solves leakage points that are caused by excessive shrinkage of marker effects. Nevertheless, these models still miss signals in some regions due to low linkage disequilibrium between the SNPs on the array used and causal variants. Thus, we discuss how such problems could be addressed by adding sequence SNPs from those regions to the prediction model.

**Conclusions:**

Residual single-marker regression analysis is a simple approach that can be used to detect regional genomic signals that are poorly captured by a model and to indicate ways to fix such problems.

**Supplementary Information:**

The online version contains supplementary material available at 10.1186/s12711-023-00830-1.

## Background

Genome-enabled prediction has been adopted as the preferred method for the prediction of breeding values in many species. Prediction accuracy (defined as the correlation between predictions and breeding values) is typically used to evaluate the performance of models used to infer breeding values, as it directly impacts response to selection. Several factors affect the accuracy of genomic predictions, including sample size, the statistical model used, and marker density [1–5]. Increasingly, genomic research and industry applications use ultra-high-density single nucleotide polymorphism (SNP) genotypes derived from either sequencing or imputation to sequence-equivalent SNP density. However, empirical evidence suggests that prediction models that include hundreds of thousands (or even millions) of SNPs do not achieve a substantially higher prediction accuracy compared to considering only a few thousand SNPs [6].

Nevertheless, measures of genomic prediction accuracy can be highly insensitive to important differences between models. For instance, models with disparate assumptions about the distribution of effects can frequently achieve similar breeding value predictions (and therefore accuracies) [7], although those models may render very different estimates of SNP effects. Furthermore, genome-wide measures of prediction accuracy may not be informative about a model’s ability to capture signals at specific genomic regions. For example, shrinkage models may perform better than variable selection procedures at capturing signals in regions of high collinearity [i.e., very high linkage disequilibrium (LD)] [8], but the latter may be better at capturing signals in regions with large-effect variants [9]. Likewise, using a higher SNP density (e.g., sequence or imputed-to-sequence genotypes) may be more important in some regions than others.

We hypothesize that the added value of ultra-high-density SNP genotypes varies substantially across genomic regions. Thus, some regions of high LD may be well covered by low- and intermediate-density arrays, while others may benefit from using a higher SNP density. However, we lack methods to identify such regions. Therefore, our first objective was to develop and evaluate a simple approach to identify genome segments that generate genetic signals that are missed by a genetic model, a phenomenon which we refer to as signal leakage. To achieve this goal, we propose a simple approach that applies single-marker regression to the residuals of the genetic model. The intuition behind the approach is straightforward: strong associations between residuals from a genetic model and individual SNP genotypes indicate genetic signals that are missed by the genetic model from which the residuals were derived.

Signal leakage may result from poor SNP coverage in a region or from model deficiencies (e.g., too much shrinkage of effects induced by a prior distribution). Therefore, our second objective was to propose a set of analyses that can shed light on the reasons why a model may fail to fully capture signals of some genome segments. To address this, we propose applying single marker regressions to residuals that are derived from different models (pedigree-based, single-step genomic best linear unbiased prediction (GBLUP), and Bayesian regressions with different prior distributions). Those analyses can be used to determine whether the signal leakage identified in a specific region is attributable to poor SNP density or to model deficiencies (e.g., excessive shrinkage).

To illustrate the methods proposed in this study, we applied residual regressions to real breeding data with a set of analyses that involved a sequence of models (pedigree, single-step GBLUP, Bayesian ridge regression, and models with variable selection priors) and different inputs for fitting the prediction model or scanning for leakage (pedigree, medium-density, and ultra-high-density SNP panels).

## Methods

Data were obtained from 39,819 pigs that belonged to a single breeding line for which average daily gain (ADG, g), ultrasound-measured backfat thickness (BF, mm), and SNP panel genotypes were available. All males were genotyped prior to phenotyping for the aforementioned traits, and all dams were genotyped prior to inclusion into the breeding herd.

### Phenotype adjustments

Before fitting genomic models, we used a larger dataset (n = 76,736, including the 39,819 animals with phenotype and genotype data, plus contemporary animals that had phenotype data only) to adjust phenotypes for the fixed effect of contemporary group (farm, sex, birth year, and week) and the random effect of common litter. The model used for precorrection included the aforementioned effects plus an animal genetic effect based on pedigree information from 1,187,225 animals. Backfat thickness was also corrected for off-test weight as a linear covariate. The pre-corrected data were then used to test for evidence of signal leakage of pedigree and genomic models.

### Genetic information

We had pedigree data for the 39,819 animals included in the study as well as medium-density (MD) and ultra-high density (UHD) SNP genotypes coded as 0,1 and 2. Missing genotypes in the latter were imputed using the AlphaImpute software [10]. For quality control, SNPs with a MAF < 0.01 and a call rate < 0.8 were removed. As a result, 41,205 SNPs remained for the MD panel. The UHD panel was based on sequence-level SNP calling. A subset of the population was directly sequenced for different depth levels, and reads were imputed using the software AlphaPeel [11]. The called variants were then imputed for the remaining population that was genotyped with the MD panel. Details of this approach are described by Ros-Freixedes et al. [12]. SNPs with a MAF < 0.001 were removed, after which ~ 18 × 10^6^ SNPs remained for further analyses.

### Training and testing sets

The study of signal leakage can be performed on training (i.e., regressing residuals on genotypes using the same sample that was used to derive those residuals) or testing data. Evaluating and comparing the leakage landscape in the training versus the testing data may be informative, for instance, in cases where the goodness-of-fit to the training data is good and prediction accuracy in the testing data is poor. In addition, comparing the leakage patterns in the testing set obtained with alternative models could provide information about the nature of the differences in predictive performance. Therefore, we divided the 39,819 animals into a training set (n = 28,156) consisting of pigs born between January 1st, 2012, and December 31st, 2016, and a testing set (n = 11,663) consisting of pigs born between January 1st, 2017, and April 30th, 2018). The Results section focuses on the analysis of the training set, but some features observed when studying the testing set are presented in the Discussion section.

### Models

We fitted five types of models to the training data (n = 28,156): pedigree BLUP (PBLUP), single-step (ss)GBLUP [13–15], Bayesian ridge regression (BRR), BayesC (i.e., a variable selection model with a point of mass at zero and a Gaussian slab) [16], and BayesB (similar to BayesC, but with a t-distribution slab) [17]. Non-genetic effects were not considered here as the data were pre-corrected for these. The sequence of models progressed from traditional pedigree models to genomic models, and from lower to higher prior density for larger SNP effects. The transition from PBLUP to ssGBLUP was smoothed by considering scenarios with incremental proportions of genomic information included in the training set (20, 50, 80, and 100% of the animals), represented as ssGBLUP_20, ssGBLUP_50, ssGBLUP_80, and ssGBLUP_100, respectively. Selection of animals with genotypes included in these scenarios was at random. In all scenarios, the genotypes used to fit these models were those from the MD SNP panel.

The PBLUP and ssGBLUP models were fitted using the BLUPF90 software family [18]. The remaining models were fitted with the BGLR R package using its default hyperparameters [19]. Briefly, for variance parameters, BGLR uses scaled inverse chi-square priors with 5 degrees of freedom and a scale parameter such that the prior expected value of the variances corresponds to a proportion of variance explained by the model equal to 0.5. For the prior proportion of non-zero marker effects, BGLR assigns a beta distribution with shape parameters equal to 5. This results in a relatively un-informative prior distribution with a prior expected value of non-zero effects equal to 0.5.

### Residual genome-wide association analysis

We used the fitted models to derive residuals (i.e., phenotypes minus predictions) for both traits. Subsequently, we tested the association between residuals and the SNP genotypes of both panels by regressing the former on the latter using single marker regression (i.e., one SNP at a time). We determined significance based on a false discovery rate (FDR) < 0.01. The association tests were conducted using the BGData R package [20] and results were displayed using Manhattan- and QQ-plots produced using the ggman [21] and ggplot2 [22] R-packages. These two displays offer different insights: Manhattan plots provide information about the distribution of significant associations across the chromosomes, while QQ-plots are more informative about the strength of the associations and the presence of p-value inflation [23].

## Results

### Signal leakage is pervasive in pedigree models

**Fig. 1:**
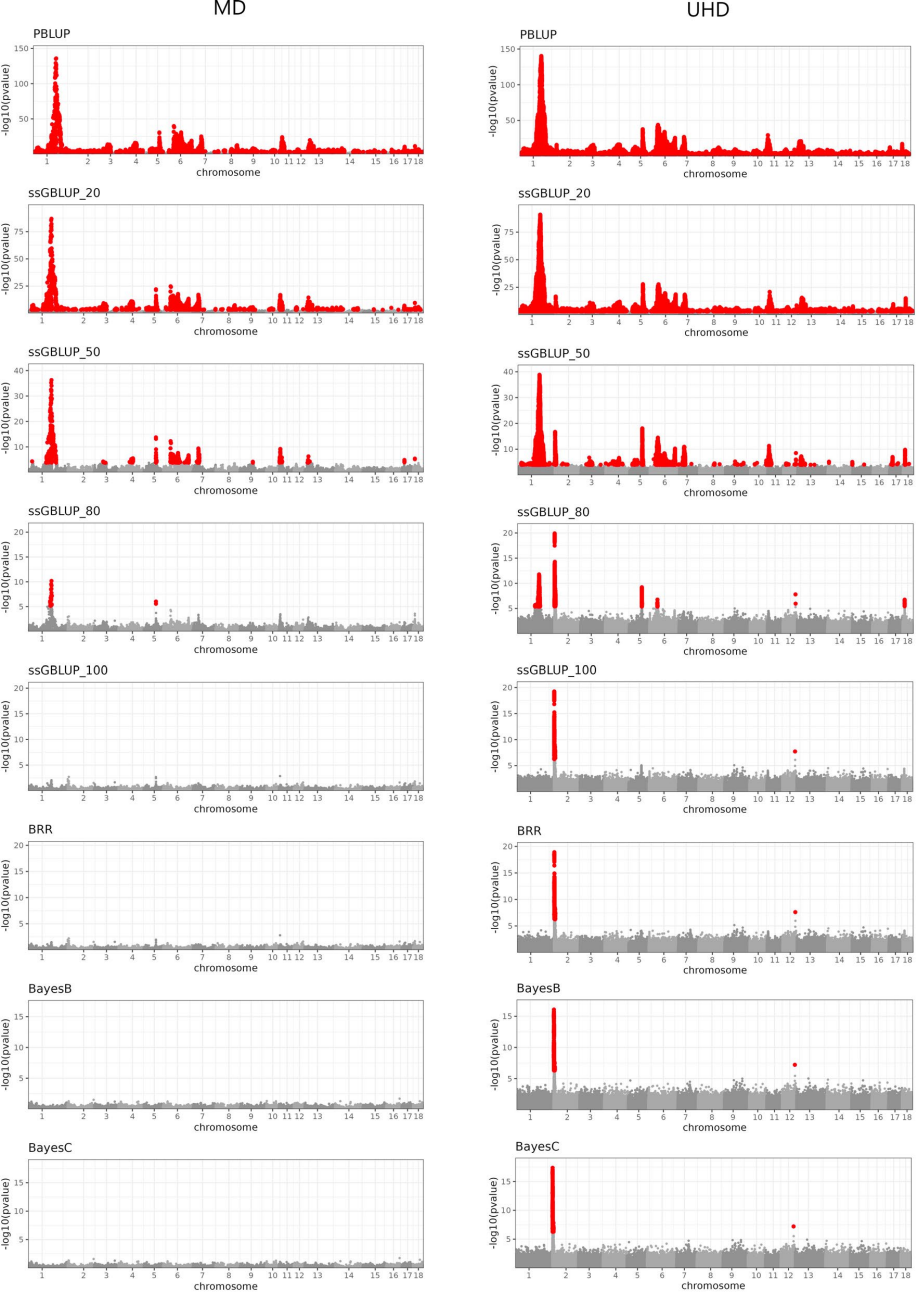
Residual association p-values (− log10 scale) for backfat thickness by model and by SNP panel. *PBLUP =* pedigree BLUP; ssGBLUP_* = single step GBLUP with *% of genotyped animals; *BRR,* *BayesB*, and *BayesC* are Bayesian models with all animals genotyped. SNP panel: *MD =* medium-density; *UHD =* ultra-high- density. SNPs with an FDR < 0.01 are highlighted in red

**Fig. 2:**
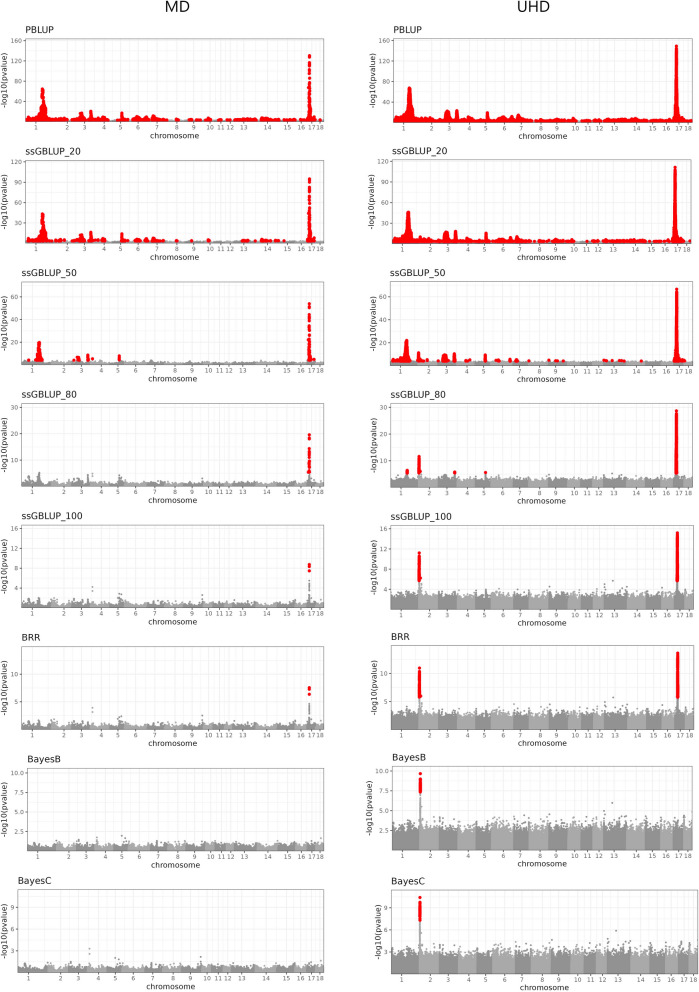
Residual association p-values (− log10 scale) for average daily gain by model and by SNP panel. *PBLUP =* pedigree BLUP; ssGBLUP_* = single step GBLUP with *% of genotyped animals; *BRR,* *BayesB*, and *BayesC* are Bayesian models with all animals genotyped. SNP panel: *MD =* medium-density; *UHD =* ultra-high density. SNPs with an FDR < 0.01 are highlighted in red

The Manhattan plots showed strong widespread signal leakage for PBLUP for both traits (Figs. 1, 2), with some regions showing stronger evidence of signal leakage than others. The Manhattan plot for the UHD variants reached maxima -log10(p) of 140.23 for BF (~ 1:161,541 kb) and 148.94 for ADG (~ 17:15,716 kb). The top associated MD variants were located in equivalent regions ~ 1:161,758 kb (− log10(p) = 135.80) and ~ 17:15,827 kb (− log10(p) = 130.36) for BF and ADG, respectively. Very few regions had no variants showing an FDR < 0.01, especially for the UHD panel. The landscapes of the associations with the MD and UHD variants were generally similar. These results indicate a large amount of local genetic signals that are missed by not including genomic information in the prediction model, and also suggest a substantial magnitude of overall missed signals. These patterns of associations likely reflect the difference in model fit (with potential consequences in predictive performance) between pedigree and genome-based models. Pedigree models are known to typically miss signals that are relevant for prediction compared to genomic models. However, we are not aware of previous attempts to characterize pedigree models in terms of (1) how the missed signals are distributed across the genome, (2) how the strength and distribution across the genome of the signals leaked by a pedigree model compare to those of genomic models, and (3) how the landscape of missed signals varies for different traits.

### The inclusion of SNP genotypes in the prediction model reduces signal leakage

Adding partial genotype information using ssGBLUP (e.g., 20% of the animals genotyped, ssGBLUP_20) reduced the extent of signal leakage; however, the significant associations between residuals and SNPs were still widespread. This suggests that the inclusion of genotypes for a relatively small proportion of individuals in ssGBLUP may not be sufficient to fully address the genome-wide signal leakage observed with pedigree models. Increasing the proportion of genotyped pigs gradually reduced the strength and extent of the residual associations and made the association peaks more local in the genome. Using 80% of the genotyped animals (ssGBLUP_80) reduced the signal leakage problem to a few regions only, for both traits and both SNP panels. Some of the regions that showed strong signal leakage with PBLUP (e.g. on Chr 1 for both BF and ADG, and Chr 6 for BF) no longer showed significant associations between SNPs and residuals with ssGBLUP_100. This was observed regardless of which SNP panel was used to evaluate the associations. However, a few significant associations remained even when 100% of the animals were genotyped. Some of these peaks were detected only with the UHD genotypes (Chr 2 for both BF and ADG), which suggests that imperfect LD between variants in the MD SNP panel and the causal variants may be a source of signal leakage for these regions. The strongest remaining leakage signal for ADG was on Chr 17. The magnitude of this peak was stronger when the UHD panel was used; however, the peak was still detected when the MD SNP panel was used, which was the same panel used to fit the ssGBLUP model; we conclude that excessive shrinkage may be the source of leakage in those cases (more information below). The patterns of the associations resulting from the residuals of BRR were very similar to those of ssGBLUP_100, and showed the same peaks for both traits, except that they were slightly stronger for the latter scenario. This resemblance was expected because both models are based on Gaussian assumptions.

### Shrinkage of estimates can contribute to signal leakage

Next, we explored whether using variable selection priors, which may reduce the extent of shrinkage of regions harboring sizable-effect variants, can resolve some of the leakage of signals that remained even when 100% of the animals were genotyped. We observed that using either the BayesB or the BayesC prior fully eliminated the strongest signal leakage peak from ssGBLUP_100 and BRR on Chr 17 for ADG. This suggests that variants of large effects, which are not fully captured when using a Gaussian prior, may be involved in this region. However, no association peaks for BF were eliminated when using a variable selection prior.

### Insufficient SNP density contributes to signal leakage in some regions

Models with variable selection prior distributions showed no leakage that could be detected when the MD panels were used to build the predictors. However, scans with the UHD panels indicated a leakage spot that was missed by the MD panel. This spot was observed at the same location for both traits (Chr 2). For ADG, this peak was hardly perceptible for the models preceding ssGBLUP_50. Thus, adding genotype data and using variable selection prior distributions were not only insufficient to solve the signal leakage, but the strength of the association was hardly affected, indicating that this peak had a different nature from that on Chr 17 for ADG. This is the behavior expected for leakage due to insufficient LD between SNPs in the MD panel and a genomic region that shows strong associations with the phenotypic trait.

### Q-Q plots

**Fig. 3:**
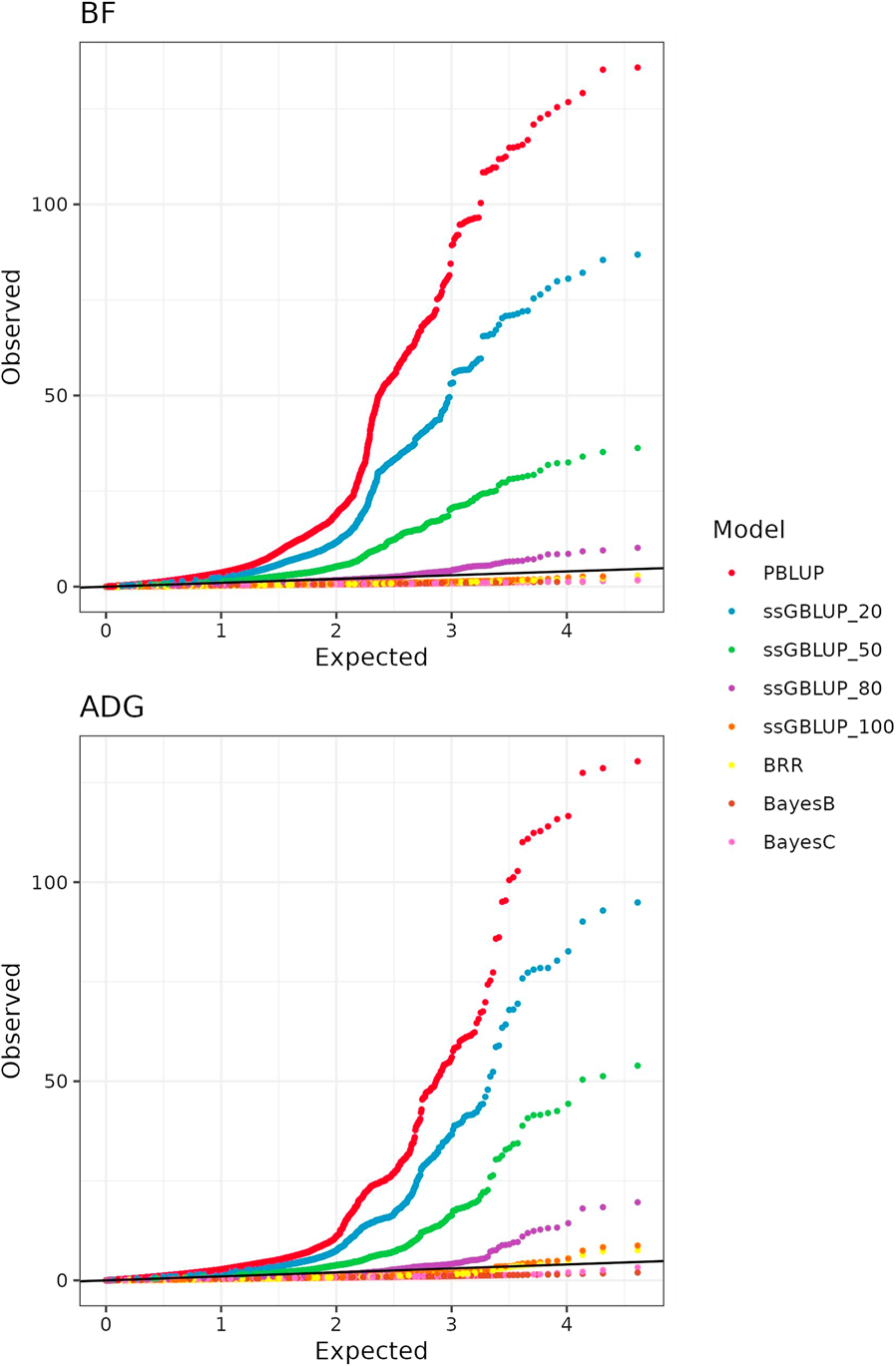
Q-Q plot of residual association p-values (− log10 scale) based on the medium-density panel for all traits (*ADG =*  average daily gain, and *BF =*  backfat thickness) and models (*PBLUP =*  pedigree BLUP; ssGBLUP_* =  single step GBLUP with *% of the animals genotyped; BRR, BayesB, and BayesC are Bayesian models with all animals genotyped)

The difference in spread and strength of signals that are missed by different models can also be examined based on QQ-plots, as shown in Fig. 3 for scans based on the MD panel. For both traits in the training set, the PBLUP model showed inflation relative to the expected values of -log10(p) starting from lower values on the x-axis, indicating genome-wide inflation. The inclusion of genotype data gradually corrected this inflation, which was still observed up to an 80% inclusion level.

## Discussion

In genomic prediction research, models are typically compared based on prediction accuracy. However, prediction accuracy can be very insensitive to important differences in the way models capture signals. Furthermore, global measures of prediction accuracy do not shed light on how a model performs for capturing local signals and how a model may be improved (e.g., by using a higher marker density in a region, or by using a different prior distribution).

To address this problem, we propose to use residual genome-wide association studies (GWAS) to identify genomic regions where genetic signals are poorly captured by a model. The results of the residual GWAS can potentially guide improvements for the genomic model from which residuals were derived (e.g., increasing the proportion of animals genotyped, increasing marker density in some regions, or using variable selection priors instead of shrinkage methods). GWAS on residuals have been used in the past (e.g. [24]), but with different objectives. The purpose of such previous residual GWAS was to map large-effect quantitative trait loci (QTL) while correcting for population structure and family effects. The method proposed here is not tailored for QTL scans, but for the evaluation and comparison of genomic prediction models by characterizing the genomic signals that these are missing.

We provided an example with a study design that allows the relative contribution of several factors to the problem of signal leakage to be disentangled. Our results suggest that, for many of the regions where leakage was detected, the use of incomplete genotype information (i.e., the use of a sizable percentage of ungenotyped animals) was a major factor contributing to signal leakage. Shrinkage of the estimates of SNP effects, which is inherent to GBLUP-type methods, and low SNP density in specific regions also appeared to be factors that contribute to signal leakage.

Several studies have considered the addition of sequence variants to commercial SNP panels to improve genomic prediction models (e.g. [25, 26]). Selection of the sequence SNPs to be used to supplement the commercial panel is typically based on direct statistical information (e.g. GWAS applied to the original trait, rather than to residuals as done here), biological information (e.g. genes that are differentially expressed or that are assigned to relevant biological pathways), or a combination of both. However, these approaches do not consider how much signal is captured/leaked by the commercial SNP panel that was used in the first place. For example, there is limited potential in adding variants that belong to a few relevant regions if leakage is spread genome-wide with little regional concentration. Alternatively, even if leakage is more local, using standard GWAS or biological information may direct us to add variants for regions from which signals are already well captured by the current panel. The residual GWAS approach presented in this study can provide an effective alternative criterion to decide how to supplement commercial SNP panels by identifying regions with strong evidence of signal leakage.

All results presented so far are based on data used as training sets for the prediction models. We also evaluated the leakage patterns in a testing set (n = 11,663). Predictions for the testing set were computed based on the models fitted in the training set, and then residuals were obtained as the difference between pre-corrected phenotypes and those predictions. The results are presented in Additional file 1 Fig. S1, Additional file 2 Fig. S2, and Additional file 3 Fig. S3. The pattern of leakage obtained with the PBLUP model in the testing set resembled the results for the same model in the training set for both traits. However, that did not apply to the other models. Similar to what we observed with the training data, using SNP genotypes also reduced the leakage in the testing data. However, as expected, for all models we had many more significant genome-wide associations in the testing dataset than in the training dataset. Such a difference likely reflects sampling variability in phenotype-genotype associations. Thus, for many regions, the models were able to fully capture the SNP-phenotype associations present in the training dataset, but such associations were slightly different in the testing dataset, thus leading to more evidence of widespread poorly captured signals. In contrast to the PBLUP model, the strongest local leakage regions that were observed in the training dataset were at least not sufficiently strong in the testing set to become noticeable above the strong widespread leakage. As a whole, these results might reflect (a) the typical lower predictive correlations in testing data in comparison with training data prediction correlations, and (b) the superiority of testing set accuracies from genomic prediction models relative to PBLUP. The latter is also reflected in the QQ-plots in Additional file 1 Fig. S3.

The residual GWAS used the standard approach which assumes that the error terms are independent and identically distributed (IID). However, Nobre and Singer [27] pointed out that estimates of residuals from linear models are not IID, even if the original error terms were IID. Nevertheless, least squares methods are known to be robust to miss-specification of assumptions about heterogeneous variances and also (co)variances of the error terms. Furthermore, most of the significant associations found in the residual GWAS disappeared when the apparent causes (e.g., high % of ungenotyped animals, use of insufficient marker density, or the use of shrinkage prior) were addressed. Nonetheless, further investigation is needed to assess whether heterogeneous variances or correlations between estimates of residuals from a model, which is used to derive them, may represent a problem.

We proposed using residual GWAS to assess the impact of the source of genetic information (pedigree vs SNPs), marker density, and prior assumptions on signal leakage. However, the same approach could be used to investigate other factors that may affect the ability of a model to capture genetic signals. For example, one could compare the leakage that results from using different imputation methods or SNP panels for genomic prediction. It may also be relevant to explore how sample size and the structure of the training dataset (family size, population structure), as well as the source of the UHD genotypes affect signal leakage. In this regard, it is worth noting that a very small sample size could lead to not detecting significant leakage simply because the data used lack the power to detect small signals that were leaked by a model. Nevertheless, this is not expected to be the case for our analysis which used larger sample sizes than what is typical for GWAS in pigs. Also, in our study, UHD genotypes were derived by combining direct whole-genome sequencing genotypes for some animals with the MD genotypes for the others. Although the imputation accuracy is reported to be very high [12], one could speculate that whole-genome sequencing of all animals may lead to detecting some additional peaks that may have been missed because of noise in the imputation.

Finally, this study focused entirely on mapping genetic signals leaked by a model; however, the same approach could be used to assess whether other omics can contribute to improving phenotypic prediction. For example, one could ask whether gene expression or methylation data can improve phenotypic prediction above and beyond what can be predicted using DNA information alone. This question could be tackled by regressing residuals from a genetic model on such omics data. Significant residual associations would indicate that some patterns that link phenotypes and these omic data are not fully captured by the genomic regression used to derive residuals. Likewise, one could use residuals from a regression of a phenotype on gene expression to investigate whether associations between gene expression and a phenotype are entirely mediated by methylation.

## Conclusions

Residual single-marker regression analysis can be used to detect genetic signals that a model fails to capture. The leakage of genetic signals is pervasive in pedigree models and ssGBLUP with a low percentage of genotyped animals. Signal leakage can also occur in genomic regressions (with all animals genotyped) due to low regional marker density or excessive shrinkage of effects. The comparison of the signals leaked by different models can shed light on the factors that lead to signal leakage and on ways to fix it.

### Supplementary Information

**Additional file 1: Figure S1.** Residual association p-values (-log10 scale) for backfat thickness in the testing set by model (PBLUP = pedigree BLUP; ssGBLUP_* = single step GBLUP with *% of genotyped animals; BRR, BayesB, and BayesC are Bayesian models with all animals genotyped) and SNP panel (MD = medium-density, UHD = ultra-high-density). SNPs with an FDR < 0.01 are highlighted in red.

**Additional file 2: Figure S2.** Residual association p-values (− log10 scale) for average daily gain in the testing set by model (PBLUP = pedigree BLUP; ssGBLUP_* = single step GBLUP with *% of genotyped animals; BRR, BayesB, and BayesC are Bayesian models with all animals genotyped) and SNP panel (MD = medium-density, UHD = ultra-high-density). SNPs with an FDR < 0.01 are highlighted in red.

**Additional file 3: Figure S3.** Q-Q plot of residual association p-values (-log10 scale) in the testing set based on the medium-density panel for all traits (ADG = average daily gain, and BF = backfat thickness) and models (PBLUP = pedigree BLUP; ssGBLUP_* = single step GBLUP with *% of genotyped animals; BRR, BayesB, and BayesC are Bayesian models with all animals genotyped).

## Data Availability

The datasets generated and analyzed in this study are derived from the PIC breeding programme and are not publicly available.
