# Supplementary figures and images for: Using residual regressions to quantify and map signal leakage in genomic prediction
Source: Genet Sel Evol. 2023 Aug 7;55:57. doi: 10.1186/s12711-023-00830-1 (PMC10405418; doi:10.1186/s12711-023-00830-1)

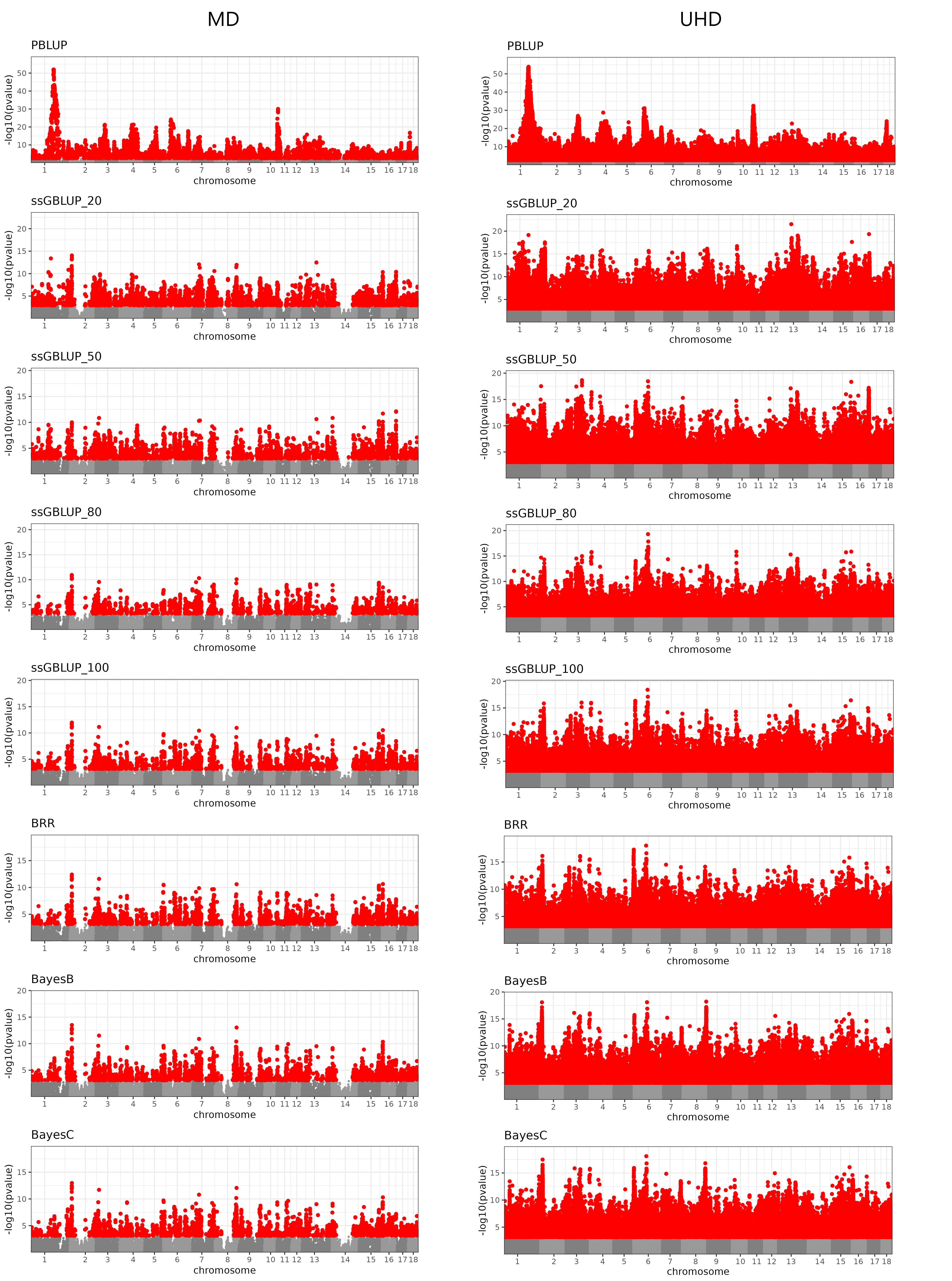

Supplement: Supplementary file 1 — Additional file 1: Figure S1. Residual association p-values (-log10 scale) for backfat thickness in the testing set by model (PBLUP = pedigree BLUP; ssGBLUP_* = single step GBLUP with *% of genotyped animals; BRR, BayesB, and BayesC are Bayesian models with all animals genotyped) and SNP panel (MD = medium-density, UHD = ultra-high-density). SNPs with an FDR < 0.01 are highlighted in red. [file 12711_2023_830_MOESM1_ESM.jpg]

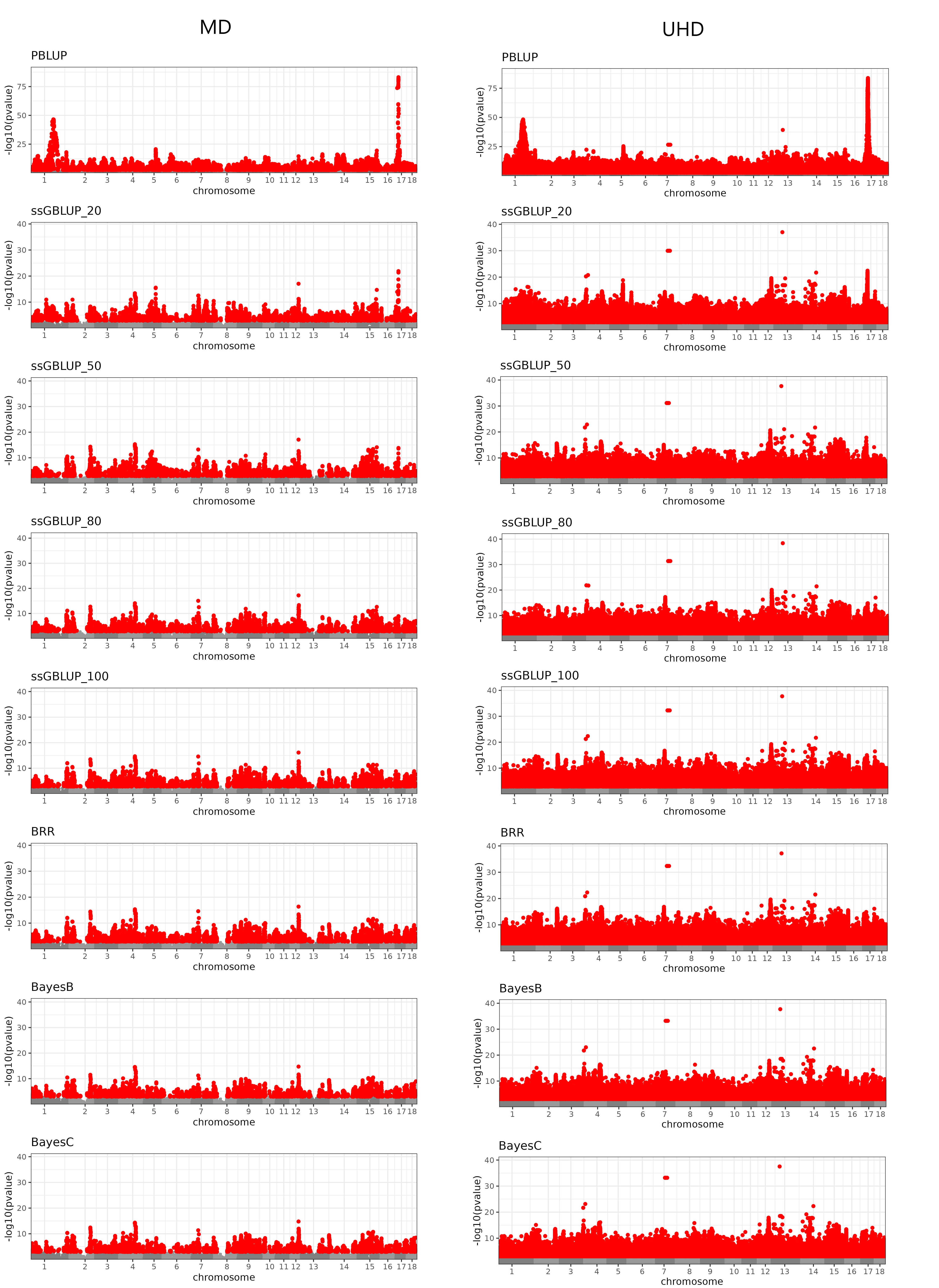

Supplement: Supplementary file 2 — Additional file 2: Figure S2. Residual association p-values (− log10 scale) for average daily gain in the testing set by model (PBLUP = pedigree BLUP; ssGBLUP_* = single step GBLUP with *% of genotyped animals; BRR, BayesB, and BayesC are Bayesian models with all animals genotyped) and SNP panel (MD = medium-density, UHD = ultra-high-density). SNPs with an FDR < 0.01 are highlighted in red. [file 12711_2023_830_MOESM2_ESM.jpg]

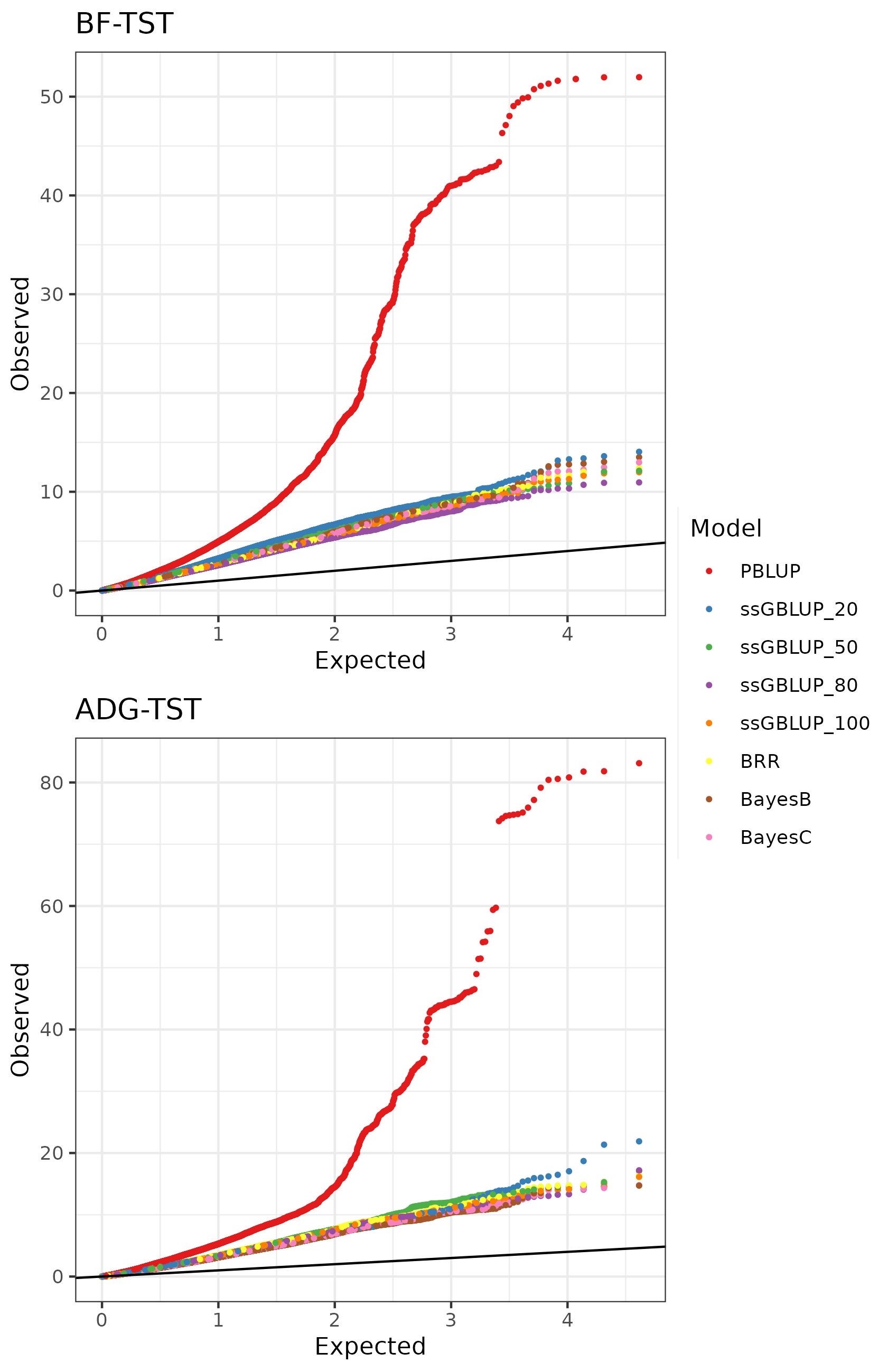

Supplement: Supplementary file 3 — Additional file 3: Figure S3. Q-Q plot of residual association p-values (-log10 scale) in the testing set based on the medium-density panel for all traits (ADG = average daily gain, and BF = backfat thickness) and models (PBLUP = pedigree BLUP; ssGBLUP_* = single step GBLUP with *% of genotyped animals; BRR, BayesB, and BayesC are Bayesian models with all animals genotyped). [file 12711_2023_830_MOESM3_ESM.jpg]
